# Self-care behaviors mediates the relationship between resilience and quality of life in breast cancer patients

**DOI:** 10.1186/s12888-022-04470-5

**Published:** 2022-12-26

**Authors:** Abbas Abdollahi, Fahad Alsaikhan, Denis Andreevich Nikolenko, Moaed E. Al-Gazally, Trias Mahmudiono, Kelly A. Allen, Bekhzod Abdullaev

**Affiliations:** 1 Department of Counseling, Faculty of Education and Psychology, Alzahra University, Tehran, Iran; 2 Department of Clinical Pharmacy, College of Pharmacy, Prince Sattam Bin Abdulaziz University, Alkharj, Kingdom of Saudi Arabia; 3 Department of Prosthetic Dentistry, Sechenov First Moscow State Medical University, Moscow, Russia; 4 College of Medicine, University of Al-Ameed, Karbala, Iraq; 5 Department of Nutrition, Faculty of Public Health, Universitas Airlangga, Surabaya, Indonesia; 6 Educational Psychology and Inclusive Education, Faculty of Education, Monash University, Clayton, Australia; 7 Department of Pathology and Physiology, Ferghana Medical Institute of Public Health, Ferghana, Uzbekistan

**Keywords:** Resilience, Self-care behaviors, Quality of life, Women, Breast cancer, Women with breast cancer, Mediator

## Abstract

Previous studies have shown that resilience could play an important role in enhancing the quality of life in women with breast cancer; however, the mediating role of self-care behaviors have not been studied. This study aims to explore the mediating role of self-care behaviors in the relationship between resilience and quality of life in breast cancer patients. A sample of 195 women with breast cancer (aged from 21 to 60 years; M = 45.32 ± 8.2) from three hospitals in Tehran, Iran completed online questionnaires measuring resilience, self-care and quality of life. The results of structural equation modeling showed that resilience (β = 0.546, *p* < .01) and self-care behaviors (β = 0.621, *p* < .01) positively predicted the quality of life in breast cancer patients. The bootstrapping analysis showed that self-care behaviors acted as a partial mediator between resilience and quality of life. The present study brings to light an underlying mechanism of the relationship between resilience and quality of life via the mediating variable of self-care behaviors for patients with breast cancer.

## Introduction

Breast cancer is prevalent worldwide with about 1.5 million women diagnosed with breast cancer annually [1]. In Iran, the prevalence of breast cancer is high in women with about 6,000 cases, of which about 1,000 breast cancer patients die annually [2]. The World Health Organization estimates the annual occurrence of breast cancer has grown by 2%. Breast cancer and the side effects of treatment, have been found to exacerbate stress, and negatively influence a patient's quality of life [3, 4]. It is, therefore, important to study quality of life and related variables in patients with breast cancer to tailor specific interventions. The present study's findings may be beneficial in planning and implementing best possible care for women with breast cancer to improve their quality of life.

Studies have shown that women with breast cancer are more likely than women without breast cancer to experience a number of factors that lead to perceptions of reduced quality of life. These factors include greater anxiety, depression [5], anger [6], aggression, hostility [7], sadness, decreased sexual function [8], dysfunctional interpersonal relationships [9], suicide ideation [10], and thoughts of mortality [11]. Quality of life is defined as a feeling of well-being that results from satisfaction or dissatisfaction with aspects of life that are important to the individual, including the interactions of physical and psychological health, spiritual wellbeing, social capital, socio-economic status, and social support [12, 13]. Therefore, people's perceptions of their quality of life, even with the same health, may be drastically different. As a result, it is often assumed that quality of life is best characterized from the perspective of the patient rather than being measured objectively.

Resilience is a psychological factor that allows individuals to not only adapt and thrive when faced with challenges or problems, but also grow and develop following stressful or aversive situations [14, 15]. Studies have indicated that resilience is a valuable resource that helps breast cancer patients maintain their quality of life during the treatment process [16], by developing psychological flexibility, a sense of meaning, and problem-solving skills [17, 18]. Conversely, low levels of resilience can diminishes a patient’s confidence to overcome stressful situations, making them feel powerless (e.g., “I cannot escape my fate”), which negatively effects the treatment process [19]. Therefore, low levels of resilience could negatively impact quality of life and high levels of resilience could be a contributing factor to experiencing a higher quality of life by breast cancer patients [20].

The mediating effect of self-care on the relationship between resilience and quality of life has yet to be studied; therefore, the present study empirically examines the mediating role of self-care behaviors. Self-care behaviors are important for women with breast cancer in the process of treatment [1, 21]. Self-care refers to preventive practices and perspectives that aid in the management and maintenance of psychological, social, and physical needs [22]. Several studies have indicated that self-care in breast cancer patients promotes physical health and well-being [23, 24]. Self-care may support individuals to regulate their emotions and lead to lowered negative emotions under difficult conditions [17] which may impact outcomes related to resiliency such as quality of life. One possible explanation for this is that individuals with higher levels of resilience are more likely to handle challenging situations (e.g., breast cancer) [14, 16] and engage in self-care behaviors, with self-care behaviors helping to promote greater health-related quality of life. People with higher levels of resilience may also be more accepting of adverse health conditions like breast cancer and be more likely to engage in strategies to reduce associated stress [25]. Additional research is needed to determine the impact of self-care on the resilience-quality of life relationship, which could lead to effective short-term and long-term interventions for women with breast cancer. Therefore, the present study examines the mediating role of self-care behaviors on the relationship between resilience and quality of life in women with breast cancer.

## Methods

### Participants

A total of 195 women with breast cancer ranging in age from 21 to 60 years (M = 45.32 ± 8.2) participated in the present study. Among the participants, 175 (90%) were married, 10 (5%) were divorced, and 10 (5%) were single. Regarding employment, 108 (55%) were full-time employees, 40 (20%) were part-time employees, and 47 (25%) reported domestic duties. Regarding education, 15 (8%) had less than a diploma, 43 (22%) had a diploma, 95 (49%) held a bachelor's degree, 36 (18%) held a master's degree, and 6 (3%) held a doctoral degree. Other demographic characteristics of the participants are shown in Table 1.

**Table 1:** Demographic data of patients with breast cancer

Variable	Number	Percentage
Types of breast cancer		
Invasive ductal carcinoma	167	85
Lobular carcinoma	11	6
Medullary carcinoma	10	5
Comedo-carcinoma	5	3
Tubular carcinoma	2	1
Breast cancer stage		
Stage I	109	56
Stage II	59	30
Stage III	17	9
Stage V	10	5

### Procedure

The study obtained ethics approval from the Human Research Ethics Committee, ________ University. Additional permission was obtained from the Iranian Ministry of Health and heads of three hospitals in Tehran. Data collection started in May 2018 and lasted for 1.5 months. All participants had previously been diagnosed with breast cancer at the hospital and their emails were given to the researchers. The researchers emailed a link to Google Forms to 310 potential participants, which included the questionnaires and a statement describing the aim of study. A total of 205 participants completed the questionnaires; 10 questionnaires were discarded due to excessive outliers. In total, 195 questionnaires were completed and used for data analysis. All participants gave informed consent before taking part. The Inclusion criteria for entering into the study included breast cancer diagnosis by breast specialists in each hospital, as well as having basic literacy skills to complete the questionnaire independently.

### Measures

#### Self-Care Utilization Questionnaire (SCUQ)

The SCUQ consists of 30 items that assess an individual’s level of self-care behaviors such as doing physical activities or talking to others when feeling stressed [26]. Questions are answered on a five-point Likert scale from 1 (*never*) to 5 (*almost always*). Overall scores range from 30 to 150 with a higher score indicating a higher level of self-care behaviors. An Iranian study on patients with breast cancer showed that the SCUQ had a good internal consistency with a Cronbach’s Alpha of 0.88 [1].

#### Connor-Davidson Resilience Scale (CDRS)

The CDRS consists of 25 items that assess an individual’s level of resilience across several dimensions, including individual competencies, self-confidence, positive acceptance of change, secure relationships, personal strength, tolerance of negative emotion, and perceived control [27]. The scale uses a five-point Likert scale from 0 (*Not at all true*) to 4 (*True nearly all the time*). The overall scores range from 0 to 100, with a higher score indicating a higher level of resilience. An Iranian study showed that the CDRS had a good internal consistency with a Cronbach’s Alpha of 0.89 [28].

### The European Organization for Research and Treatment of Cancer Quality of Life Questionnaire (EORTC QLQ-C30)

The EORTC QLQ-C30 consists of 30 items that assess quality of life among cancer patients. Items on the scale measure physical, cognitive, and social functioning. The scale uses a four-point Likert scale, with the exception of the 2 items of the quality of life scale that use a seven-point Likert scale from 1 (*very poor*) to 7 (*excellent*) [29]. A higher score on the scale suggests greater functioning. A higher score on the symptom measures suggests a higher level of symptom burden (In this study, the items were reversed and a higher score indicated a lower level of symptoms). The EORTC QLQ-C30 was validated among Iranian breast cancer patients with an excellent reliability [30].

### Statistical method

Variance-based structural equation modeling using Smart-PLS 3 software (version 3.2.3) was used to answer the research hypotheses in this study [31]. The partial least square approach was used as it has the advantages of being able to analyze the model with a small sample size, is less sensitive to non-normal data, is capable of assessing complex models, and allows for mediation analyses [32].

## Results

### Measurement model

Average Variance Extracted (AVE) was calculated to measure convergent validity and Composite Reliability (CR) and Cronbach's alpha were calculated to assess convergent reliability. As seen in Table 2, the values of AVE, CR, and Cronbach's alpha were larger than the cut-off scores of 0.5, 0.7, and 0.7, respectively, showing suitable convergent validity and reliability [33].

**Table 2:** Values of composite reliability, average variance extracted, heterotrait-monotrait ratio, variance inflation factor, mean, and standard deviation

Variable	Cronbach's alpha	CR^a^	AVE^b^	HTMT^c^	VIF^d^	M	SD
Self-Care	0.88	0.83	0.7	0.71	1.21	93	9.12
Resilience	0.89	0.84	0.8	0.70	1.34	62	4.16
Quality of life	0.87	0.82	0.7	0.70	1.67	56	5.76

^a^Composite Reliability, ^b^Average Variance Extracted, ^c^Heterotrait-Monotrait Ratio, and ^d^Variance Inflation Factor

The Heterotrait-Monotrait Ratio (HTMT) and the Variance Inflation Factor (VIF) were calculated to assess discriminant validity and multicollinearity, respectively. As seen in Table 2, the values of HTMT and VIF were less than the cut-off scores of 0.85 and 5, respectively, suggesting sufficient discriminant validity [34].

### Structural model

The hypotheses, and coefficient of determination (R^2^), effect size (f^2^), and Stone-Geisser (Q2) values were all examined at the structural model stage. The results from the structural model showed resilience (β = 0.546, *t* = 6.34, *p* < 0.001) and self-care behaviors (β = 0.621, *t* = 7.11, *p* < 0.001) positively predicted quality of life. To assess the variance in quality of life by resilience and self-care behaviors, the coefficient of determination (R^2^) was used. R^2^ for quality of life was 0.514, revealing that resilience and self-care behaviors explained 51.4% of the changes in breast cancer patients’ quality of lives. This value was greater than the cut-off score value of 0.35 that is considered a moderate coefficient of determination [34]. The effect size (f^2^) was used to explore the effect of each predictor variable on the criterion variable. Effect sizes (f^2^) for resilience and self-care behaviors were 0.38 and 0.39, respectively, indicating large effect sizes (> 0.35) to explain quality of life [35]. The Stone-Geisser (Q^2^) value was 0.38, indicating large predictive relevance of breast cancer patients’ quality of lives [34].

### Mediating effect of self-care behaviors

**Fig. 1:**
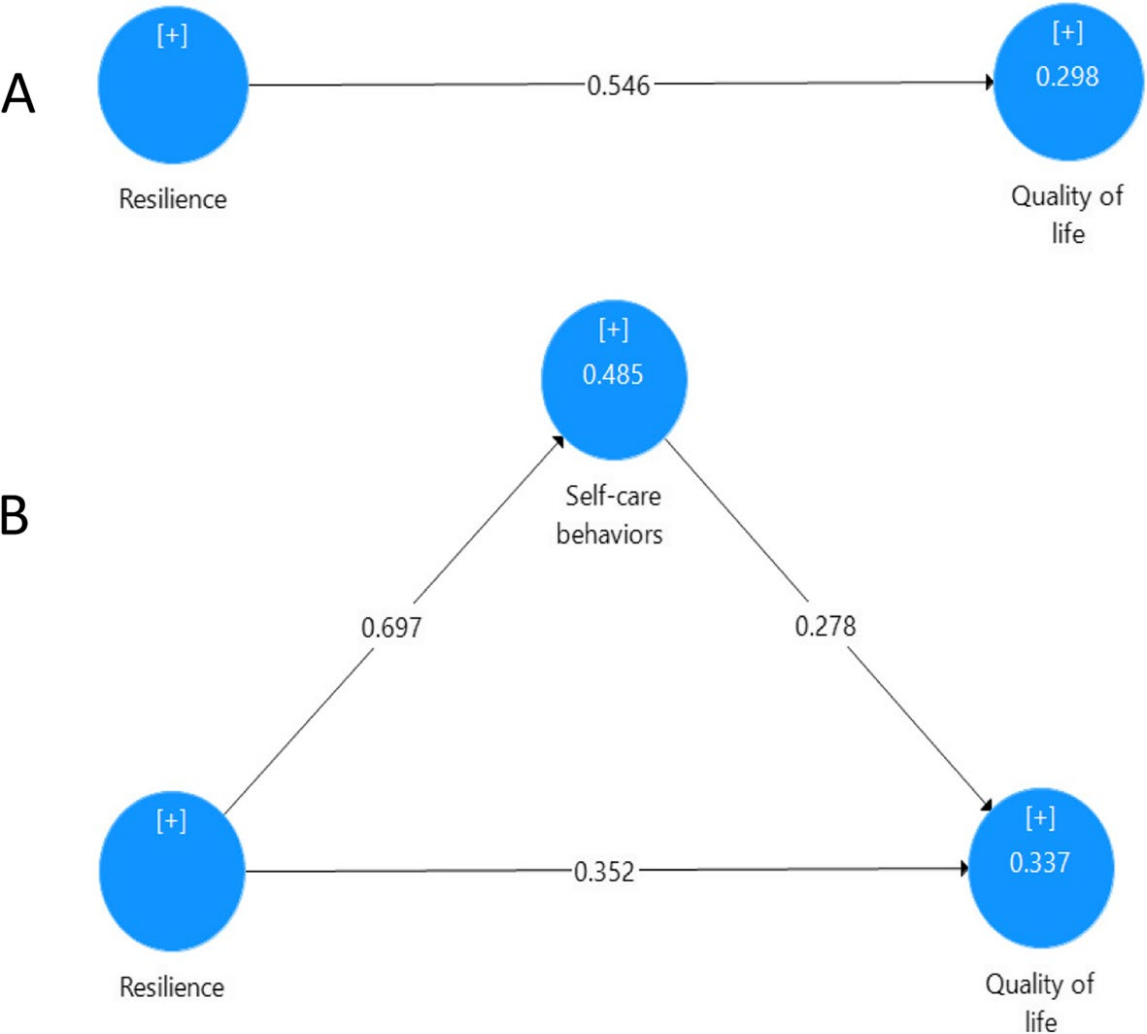
**A** Illustration of a direct effect (resilience affects quality of life). **B** Illustration of a mediation model. (resilience is hypothesized to exert an indirect effect on quality of life through self-car behaviors)

A bootstrapping approach (*n* = 5,000, α = 0.05 and CI = 95%) in smart PLS 3 software was employed to test the mediating role of self-care behaviors in the relationship between resilience and quality of life. The mediating effect of self-care behaviors is confirmed when the zero is outside the CI in the full mediation model [36]. In the direct model, the path between resilience and quality of life was significant (β = 0.546, *t* = 6.34, *p* < 0.001) (see Fig. 1). In the full mediation model, when self-care behaviors as a mediating variable was added, the β coefficient between resilience and quality of life was decreased; however, the path was still significant (β = 0.352, *t* = 3.41, *p* < 0.001). Therefore, self-care behaviors partially mediated the relationship between resilience and quality of life. Variance Accounted For (VAF) was calculated to estimate the effect size of mediating variable of self-care behaviors in the relationship between resilience and quality of life. The value range of VIF is between zero and one, and the closer value of one indicating the greater effect size of the mediating variable. A value of AVF was 0.36 and this value is in the range of 0.2 to 0.8 is considered as a moderate effect size [37].

### Discussion

The first goal of this study was to examine the relationships between resilience, self-care behaviors, and quality of life in breast cancer patients. Resilience was found to be a major positive predictor of quality of life in breast cancer patients in the study. A possible explanation for the positive relationship between resilience and quality of life in breast cancer patients is that individuals with high levels of resilience are more likely to use effective problem-solving strategies under stressful situations, generate positive emotions, diminish negative emotions, promote social behaviors to receive social support, and manage their new circumstances [16].

The second goal of this study was to examine the mediating role of self-care behaviors in the link between resilience and quality of life. The findings of this study showed that self-care behaviors partially mediate the relationship between resilience and quality of life. That is, resilience in patients with breast cancer may increase health-related behaviors and may lead patients to experience a greater quality of life. A possible explanation is that resilience may diminish negative feelings, such as distress and anger, leading to health-related behaviors (e.g. enough sleep and rest, physical activity, and personal hygiene), ultimately improving their health and contributing to better quality of life (Zhang et al., 2017). The findings support previous studies and indicate that resilience is one of the important factors in increasing health-related behaviors. Positive heath behaviors help breast cancer patients manage stress and help accelerate recovery in their treatment, and helps patients experience a higher quality of life [17, 18].

The results of the present study showed that resilience may lead to self-care behavior and such behaviors help people take better physical and psychological care of themselves and ultimately experience a higher level of quality of life. Psychologists, nurses, and physicians could help patients by increasing their resilience to engage in self-care behaviors in order to improve their physical and mental health, and quality of life. It is possible that by increasing resilience, self-care behaviors in breast cancer patients would increase, and they may experience greater quality of life.

Data were collected from a small number of women with breast cancer in three hospitals in Tehran and could not be generalized to other breast cancer patients in other geographical areas. The oldest participant in this study was 60 years old. It is possible that women with breast cancer older than 60 years did not have access to technology or literacy to participate in this study. Future studies could employ other methods for data collection, such as interviews, to obtain information from breast cancer patients older than 60 years.

The findings of this study indicated that resilience contributes to self-care behaviors and such behaviors help to improve the quality of life in patients with breast cancer.

## Data Availability

The data is available in fighshare repository website with https://doi.org/10.6084/m9.figshare.9413231
